# Profiling of Low-Molecular-Weight Carbonyls and Protein Modifications in Flavored Milk

**DOI:** 10.3390/antiox9111169

**Published:** 2020-11-23

**Authors:** Michele Wölk, Theres Schröter, Ralf Hoffmann, Sanja Milkovska-Stamenova

**Affiliations:** 1 Institute of Bioanalytical Chemistry, Faculty of Chemistry and Mineralogy, Universität Leipzig, 04103 Leipzig, Germany; michele.woelk@uni-leipzig.de (M.W.); Ralf.hoffmann@bbz.uni-leipzig.de (R.H.); 2 Center for Biotechnology and Biomedicine, Universität Leipzig, 04103 Leipzig, Germany; 3 Kohrener Landmolkerei GmbH, 09322 Penig, Germany; schroeter@kohrener-landmolkerei.de

**Keywords:** Maillard reaction, oxidation/carbonylation, protein modifications, low molecular weight carbonyls, flavored milk

## Abstract

Thermal treatments of dairy products favor oxidations, Maillard reactions, and the formation of sugar or lipid oxidation products. Additives including flavorings might enhance these reactions or even induce further reactions. Here we aimed to characterize protein modifications in four flavored milk drinks using samples along the production chain—raw milk, pasteurization, mixing with flavorings, heat treatment, and the commercial product. Therefore, milk samples were analyzed using a bottom up proteomics approach and a combination of data-independent (MS^E^) and data-dependent acquisition methods (DDA). Twenty-one small carbonylated lipids were identified by shotgun lipidomics triggering 13 protein modifications. Additionally, two Amadori products, 12 advanced glycation end products (AGEs), and 12 oxidation-related modifications were targeted at the protein level. The most common modifications were lactosylation, formylation, and carboxymethylation. The numbers and distribution of modification sites present in raw milk remained stable after pasteurization and mixing with flavorings, while the final heat treatment significantly increased lactosylation and hexosylation in qualitative and quantitative terms. The processing steps did not significantly affect the numbers of AGE-modified, oxidized/carbonylated, and lipid-carbonylated sites in proteins.

## 1. Introduction

Bovine milk is an important food in human nutrition as it can be converted into a wide range of products, such as yoghurt, cheese, and various drink types [1]. Raw milk is typically heat-treated to increase the microbiological safety and shelf-life [2]. In 2017 around 99% of the raw milk produced in the European Union (EU) was processed with ~11% used to produce milk drinks [3]. Commonly-applied procedures are high-temperature, short-time pasteurization (HTST, ≥72 °C for at least 15 s) as a rather mild treatment, and ultra-high temperature (UHT) treatment (≥135 °C up to 4 s) [2,4]. Independent of the treatment, the addition of flavorings, such as chocolate, strawberry, or vanilla, is getting more popular. This requires an additional manufacturing step, i.e., addition of flavorings and other ingredients, followed by a final heat treatment prior to packaging and transportation of the products. 

**Figure 1:**
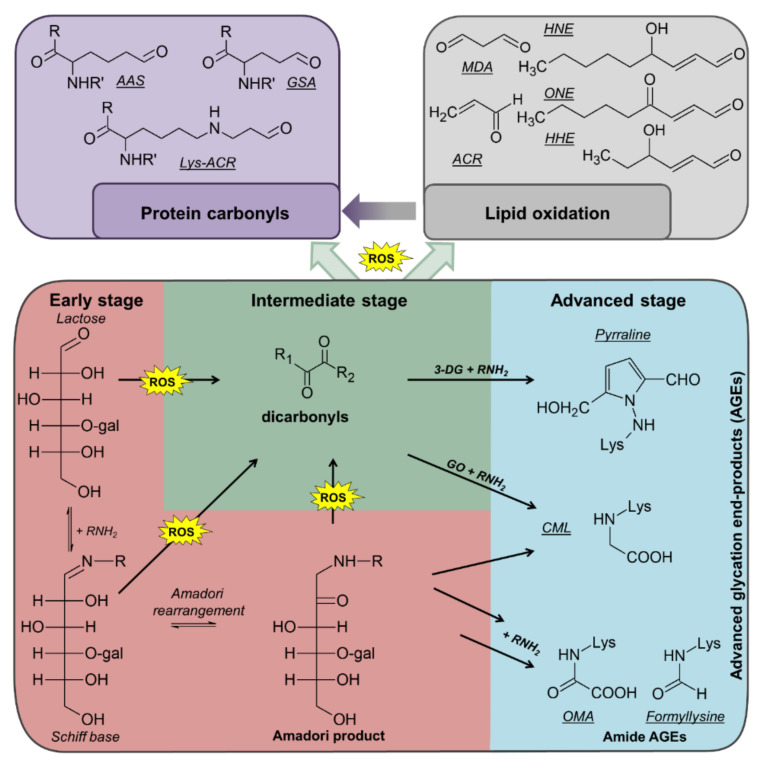
Overview of reactions that can occur during thermal processing of milk. ROS, reactive oxygen species; gal, galactose; OMA, oxalic acid monolysinylamide; CML, carboxymethyllysine; GO, glyoxal; 3 DG, 3-deoxyglucosone; ACR acrolein; MDA, malondialdehyde; HHE, 4-hydroxy-2-hexenal; HNE, for 4-hydroxy-2-nonenal; ONE, 4-oxo-2-nonenal; AAS, 2 amino-adipic-semialdehyde; GSA, glutamic-semialdehyde.

During the multiple processing steps and subsequent storage, diverse reactions can occur. Proteins, lipids, and sugars can be oxidized with sugars as well as sugar- and lipid-oxidation products being able to modify proteins yielding various post-translational modifications (PTMs) (Figure 1) [5,6,7,8]. For example, amino groups in proteins, especially the ε-amino group of lysine residues, can react with reducing sugars (mostly lactose) yielding Amadori products (lactulosyllysine) [7,8]. These relatively-stable intermediates can form advanced glycation end products (AGEs) in consecutive reactions, which also generate dicarbonyl species, such as 3-deoxyglucosone (3-DG), methylglyoxal (MGO), and glyoxal (GO), by different pathways (Figure 1) [6,7]. Dicarbonyl compounds can also derive from metal-catalyzed oxidation (MCO) of polyunsaturated fatty acids (PUFAs) [9]. Due to high reactivity, dicarbonyls can modify proteins producing different AGEs and protein-bound carbonyls, such as pyrraline (from 3-DG), argpyrimidine (from MGO), and N^ε^-carboxymethyllysine (CML, from GO) [6,10,11,12]. Protein carbonyls are also formed by MCO of lysine (2-amino-adipic-semialdehyde, AAS) or arginine and proline residues (glutamic-semialdehyde, GSA) or from lipid peroxidation products (LPPs), such as 4-hydroxy-2-nonenal (HNE), 4-oxo-2-nonenal (ONE), 4-hydroxy-2-hexenal (HHE), malondialdehyde (MDA), or acrolein (ACR) yielding advanced lipoxidation end products (ALEs) at lysine, cysteine, and histidine residues [5,13,14]. 

These protein modifications influence the nutritional and functional properties of milk [5,7,8]. For example, glycated lysine residues reduce the protein nutritional value, while the Maillard reactions lead to desired or undesired changes in color and aroma [7,8]. Lipid oxidation and formation of ALEs significantly reduce food quality when products with high fat content are processed and stored, such as inducing rancid flavors and color changes [5]. Additionally, volatile aldehydes and ketones formed during heating of milk, contribute to the typical aroma of UHT-treated milk [15,16].

Recently, glycation was extensively studied in raw, pasteurized, and UHT milk, and infant formula [10,11,17,18,19,20,21]. The numbers and quantities of lactose- and hexose-derived Amadori products increased from raw to pasteurized to UHT milk and further upon storage [18,19,20]. Harsher processing increased the numbers of identified AGEs, with formyllysine (FL) and CML representing the main AGEs present in milk [21]. Drusch and coworkers showed, by an HPLC of hydrolysates, that CML content varies among different milk products including cocoa and other flavored milks [12]. Among the flavored milk products, CML was detectable mainly in cocoa samples. Thus, the authors proposed that Amadori products had already formed during the production of cocoa powder [12]. Protein carbonylation also increased from raw to pasteurized and UHT milk [22,23]. 

Furthermore, volatile compounds including aldehydes were quantified in milk samples and pasteurization increased their content [15,16,24]. Several aldehydes, such as hexanal and nonanal, were detected at higher concentrations in UHT milks containing higher fat content (3% vs. 1%) [15]. The concentrations of volatile compounds also depend on the fodder, as they can diffuse into the milk [25,26]. Volatile aldehydes are known to form modified protein species, but this has not been considered for milk proteomic studies. 

In the current study, we profiled small carbonyl species, i.e., low molecular weight (LMW) carbonylated lipids, and targeted the corresponding 13 PTMs together with 26 PTMs derived from glycation, AGEs, and carbonylation/oxidation in the proteome of cocoa-, vanilla-, strawberry-, and chocolate-flavored milk drinks. The influence of processing steps and the addition of flavorings was studied in samples collected after pasteurization, mixing with flavorings, final heat treatment, and shipment to a regional store, relative to the original raw milk sample. Drinks differed in flavorings, fat content (1.5% or 3.5%), and final heat processing steps (HTST or UHT). Irrespective of the type of flavoring, all drinks showed similar increases in the numbers and quantities of lactosylated (Lac) peptides, which occurred mostly at the final heating step, while all other PTMs were only slightly affected. 

## 2. Materials and Methods 

### 2.1. Reagents

Sodium deoxycholate (≥97%), trichloroethanol (≥99%), tris-(-2-carboxyethyl)phosphine (TCEP, ≥98%), 7-(diethylamino)coumarin-3-carbohydrazide (CHH, ≥98%), and *tert-*butyl methyl ether (MTBE, ≥99.8%) were purchased from Sigma Aldrich Chemie GmbH (Taufkirchen, Germany). Acrylamide/bis solution (30% *w/v*), bovine serum albumin (BSA), ammoniumpersulfate, Coomassie Brilliant blue G250, tetraethylene diamine (>98.5%), and thiourea (>99%) were obtained from SERVA electrophoresis GmbH (Heidelberg, Germany). Urea (>99.5%), sodium dodecyl sulfate (SDS, >99.5%), dithiothreitol (DTT, >99%), and glycerol (>99.5%) were purchased from Carl Roth GmbH & Co. KG (Karlsruhe, Germany). Acetonitrile (ULC-MS grade, >99.97%), methanol (ULC-MS grade, >99.97%), dimethylformamide (DMF, 99.8%), ammonium formate (ULC-MS grade), and formic acid (ULC-MS grade, >99%) were obtained from Biosolve B.V. (Valkenswaald, Netherlands). Ammoniumbicarbonate (>99.0%) and β-mercaptoethanol were purchased from Fluka (Buchs, Germany). Chloroform (≥99.8%) was obtained from Merck KgaA (Darmstadt, Germany). Iodoacetamide (IAA, ≥99%) and tris ultrapure (≥99.9%) were purchased from AppliChem GmbH (Darmstadt, Germany). Sequencing-grade modified trypsin was obtained from Promega GmbH (Mannheim, Germany). Water (resistance R > 18 mΩ/cm; total organic content < 10 ppb) was purified by a PureLab Ultra Analytic system (ELGA Lab Water, Celle, Germany).

### 2.2. Milk Samples

Cocoa-, vanilla-, chocolate-, and strawberry-flavored milk drinks and samples from their processing steps were obtained from a local dairy company. Milk was processed as follows: raw milk was heated to 55 °C and skimmed by a centrifugal separator, then the fat content was adjusted to the desired value, and the milk was pasteurized (at least 72.5 °C for minimum 15 s). Next, ingredients such as flavorings were added and a final heat treatment was applied (second pasteurization or UHT-treatment at 140 °C for 3 s). Samples were collected at different processing steps—raw milk, pasteurized milk, milk mixed with flavorings, and the final milk drink obtained after a second pasteurization (cocoa drink, 1.5% fat) or UHT (vanilla, 1.5% fat; chocolate 3.5% fat; strawberry, 3.5% fat). Additionally, the vanilla, chocolate, and strawberry UHT drinks were bought in a local supermarket. Based on the manufacturer’s information provided on the products, the following ingredients were added to the milk drinks: for cocoa 1.4% low fat cocoa powder and carrageenan; for vanilla 0.5% Bourbon vanilla, beta carotene, and carrageenan; for chocolate 7.3% (Belgian) chocolate, sugar, and carrageenan; and for strawberry 3.5% strawberry juice from strawberry juice concentrate, aroma, and carmine. The sugar content was 88 g/L for cocoa, 104 g/L for vanilla, 100 g/L for chocolate, and 96 g/L for strawberry drinks.

### 2.3. Derivatization, Extraction, and MS Analysis of Carbonylated Lipid Species

Samples collected after final processing of each milk drink (50 µL) were incubated with CHH (0.1 mol/L in DMF, 1 h, 37 °C) to derivatize carbonyl-containing LPP products (oxoLPPs) [27]. Lipids were extracted with methanol (350 µL) and MTBE (1.25 mL) [19]. After incubation (1 h, 4 °C) water (315 µL) was added and the samples were incubated again (10 min, 4 °C). The samples were centrifuged (10 min, 10,000× *g*) and the organic phases were collected. The aqueous phases were washed with a mixture of MTBE:methanol:water (500 µL, 4:1.2:1, *v/v/v*) and centrifuged (10 min, 10,000 *g*). The organic phases were combined and dried under vacuum. 

Derivatized samples reconstituted in ESI buffer (5 mmol/L ammonium formate in a mixture of methanol and chloroform (2:1, *v/v*) were analyzed by direct infusion (15 µL) using a robotic nanoflow ion source (TriVersa NanoMate, Advion, Ithaca, NY) and nanoelectrospray chips (1.3 kV ionization voltage) coupled to an LTQ Orbitrap XL ETD mass spectrometer (Thermo Fisher Scientific, Bremen, Germany) at a targeted mass resolution of 100,000 at *m/z* 400 in positive ion mode. Tandem mass spectra were recorded in data-dependent acquisition (DDA) for the five most intense signals (top 5) using collision-induced dissociation (CID). Due to the high complexity of the sample, gas phase fractionation (GPF) was applied utilizing five overlapping *m/z* segments (*m/z* 310–355, 350–405, 400–485, 480–605, and 600–710) for 3 min corresponding to a total analysis time of 15 min. The isolation window for segments 1, 2, and 5 was set to 1 *m/z* unit and for segments 3 and 4 to 1.5 *m/z* units. Precursors fragmented five times within 10 s, were excluded for fragmentation for 1 min. CHH-derivatized species were manually annotated based on their fragment spectra (Figure S1) as described by Milic et al. [28,29]. Identified species were integrated into the targeted analysis.

### 2.4. Extraction and Digestion of Proteins

Proteins were extracted from three aliquots of each milk drink sample (50 µL) using a mixture of methanol, chloroform, and water (1750 µL, 1:2:1.7, *v/v/v*) [19]. The protein pellet was dried under vacuum and dissolved in lysis buffer (7 mol/L urea, 2 mol/L thiourea, 50 mmol/L Tris-HCl, pH 7.5). Protein concentrations were determined by a Bradford assay using a serial dilution of BSA for calibration and confirmed by the band intensities in SDS-PAGE [19]. Thus, protein samples (100 µg) were diluted with ammonium bicarbonate (25 mmol/L) to a final concentration of 1 g/L [22]. Proteins were denatured by adding sodium deoxycholate (1% *w/v*), reduced with TCEP (5 mmol/L, 60 °C, 30 min, 550 rpm), alkylated with IAA (10 mmol/L, 37 °C, 30 min, 550 rpm, in darkness), the excess of IAA quenched with DTT (10 mmol/L, 37 °C, 30 min, 550 rpm), and trypsin added (2 µg). The samples were digested overnight (37 °C, 550 rpm) and acidified with formic acid (0.5% *v/v*). Precipitated sodium deoxycholate was removed by centrifugation (10 min, 9700× *g*). The supernatant was desalted by solid phase extraction (SPE; Oasis HLB 1cc, 30 mg, Waters GmbH, Eschborn, Germany) and dried under vacuum [19].

### 2.5. Peptide Analysis

The dried digests were reconstituted in aqueous acetonitrile (3%, *v/v*) containing formic acid (0.1%, *v/v*). Aliquots of the three samples of each milk drink were pooled (175 ng, 10 µL) and analyzed on a nanoAcquity UPLC (Waters GmbH) coupled on-line to a Synapt G2-S*i* mass spectrometer (Waters GmbH) equipped with a nanoESI source (Waters GmbH). Eluents A and B were water and acetonitrile, respectively, both containing formic acid (0.1%, *v/v*). Peptides were trapped on a nanoAcquity Symmetry C18-column (internal diameter (ID) 180 μm, length 2 cm, particle diameter 5 μm) at a flow rate of 5 μL/min (3% eluent B) and separated on a BEH 130 column (C18-phase, ID 75 μm, length 10 cm, particle diameter 1.7 μm) using a flow rate of 0.35 μL/min and a column temperature of 35 °C. Peptides were eluted by linear gradients from 3 to 40% eluent B within 89 min and to 85% eluent B within 5 min. Mass spectra were recorded in positive ion mode using data-independent acquisition (DIA, MS^E^) and DDA modes with scan times of 0.5 s and 0.2 s, respectively [30]. CID was induced in the trap cell using a collision energy ramp (18–50 V for MS^E^ and 25–50 V for DDA). GluFib (100 fmol/µL in 50% (*v/v*) aqueous acetonitrile containing 0.1% (*v/v*) formic acid) was used as a lock mass reference in intervals of 30 s. Tandem mass spectra were acquired in DDA mode for the five most intense precursor ions using scan times of 0.4 s.

Pools and individual digests were analyzed further on a nanoAcquity UPLC (Waters GmbH) coupled on-line to an LTQ Orbitrap XL ETD mass spectrometer equipped with a nano-ESI source (Thermo Fisher Scientific, Bremen, Germany). Peptides were trapped (nanoAcquity Symmetry C18-column) at a flow rate of 5 µL/min (3% eluent B) and separated on a BEH 130 column (30 °C) using a flow rate of 0.4 µL/min and the gradient described above. The transfer capillary temperature was set to 200 °C and an ion spray voltage of 1.4 kV was applied to a PicoTip^TM^ on-line nano-ESI emitter (New Objective, Berlin, Germany). Mass spectra (*m/z* range 400 to 2000) were recorded in the Orbitrap mass analyzer at a resolution of 60,000 at *m/z* 400. For pooled samples tandem mass spectra were acquired using DDA for the six most intense signals in CID mode (isolation width of 2 *m/z* units, normalized collision energy of 35%, activation time of 30 s, default charge state of 2, intensity threshold of 500 counts, dynamic exclusion window of 60 s) using a retention-time based (±1.5 min) exclusion list of unmodified peptides identified by DIA. For individual samples tandem mass spectra were acquired using electron transfer dissociation (ETD, isolation width of 2 *m/z* units, activation time 100 ms, default charge state 2, intensity threshold of 500 counts, dynamic exclusion window of 60 s) in DDA mode for the six most intense signals using an inclusion list of all proposed modified peptides.

### 2.6. Data Analysis

MS^E^ spectra were searched against the SwissProt database (bovine proteins, release 2016_11) using Progenesis QI for proteomics (Version 4.0, Waters) to create an exclusion list of unmodified peptides. Parameters were low energy threshold 150 counts and elevated energy threshold 30 counts. Oxidation of Met and carbamidomethylation on Cys (Cam) were set as variable modifications. Unmodified peptides with a peptide score above 4 were used to generate an exclusion list specific for each pooled milk drink. Data acquired in DDA mode on the Synapt G2-S*i* were used to correct the retention time between instruments.

Data acquired on the LTQ Orbitrap XL ETD were searched using Sequest within Proteome Discoverer 1.4 (Thermo Fisher Scientific, Bremen, Germany) applying the following parameters—bovine milk database (release 2016_11), trypsin as protease, maximum three missed cleavage sites, precursor mass tolerance of 10 ppm, fragment mass tolerance of 0.8 Da, and dynamic modifications. As a template allows only up to six dynamic modifications, the search was divided into eleven templates—one for Amadori products, three for AGE-modifications, three for oxidation/carbonylation products (MCO-derived products and LPP adducts), and four for LMW carbonyl adducts with all templates also containing Cam (+57.02 Da, C) and methionine oxidation (Ox, +15.995 Da, M). All targeted modifications are listed in Table S1. It should be noted that LMW carbonyl adducts include only PTMs derived from small aldehydes (carbonylated lipids) identified in the organic phases of milk extracts. These compounds do not produce protein-bound carbonyls and should be distinguished from LPP adducts considered as carbonylation modifications (Table S1). 

Each milk pool was analyzed by DDA (CID mode) using an exclusion list generated from the MS^E^ data using adjusted retention times and a retention time window of ±1.5 min. The acquired tandem mass spectra were searched with Sequest using Proteome Discoverer (as described above). One inclusion list was generated by combining all modified peptides identified with high or medium confidence, i.e., ranked on position one and charge-state-specific scores (Xcorr ≥ 2 for z = 1.5, ≥ 1.9 for z = 3, ≥ 2.8 for z = 4, and ≥ 3.8 for z = 5), and previously identified in bovine milk [18,19,21]. All individual milk samples were analyzed (DDA in ETD mode) using the generated inclusion list (retention time window of ±2 min) and searching the recorded tandem mass spectra as described above. Modified peptides identified by both analyses were considered for relative quantification after manual confirmation of each tandem mass spectrum. Quantification relied on the peak areas of the corresponding extracted ion current chromatograms (XICs) calculated by Skyline (19.1.0.1093 MacCoss, Seattle, WA, USA). The relative quantities calculated for a modified peptide were normalized by dividing the peak area by the sum of the peak areas of several unmodified peptides corresponding to the same protein, i.e., seven peptides for β-lactoglobulin, six peptides for α_S1_-casein, and eleven peptides for α_S2_-casein. The calculated peak area ratio was normalized to the highest observed ratio in any of the samples (100%).

## 3. Results

### 3.1. Identification of Modified Peptides

**Figure 2:**
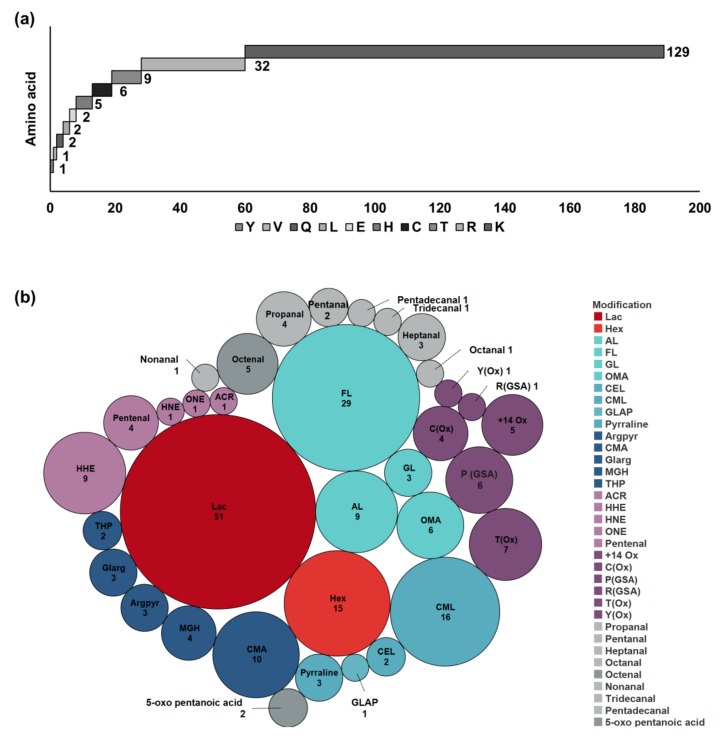
(**a**) Number of unique modified amino acid residues and (**b**) peptides carrying Amadori (red), advanced glycation end product (AGE) (blue), carbonylation/oxidation (purple), and low molecular weight (LMW) carbonyl (grey) modifications. Doubly-modified peptides were counted once for each modification. The following modifications were identified: hexosylation (Hex), lactosylation (Lac), acetyllysine (AL), formyllysine (FL), glycerinyllysine (GL), oxalic acid monolysinylamide (OMA), carboxyethyllysine (CEL), carboxymethylation of Lys/Arg (CML/CMA), glyceraldehyde-derived pyridinium (GLAP), pyrraline, argpyrimidine (Argpyr), glyoxal-derived hydroimidazolium (Glarg), methylglyoxal-derived hydroimidazolones (MGH), tetrahydropyrimidine (THP), acrolein (ACR), 4-hydroxy-2-hexenal (HHE), 4-hydroxy-2-nonenal (HNE), 4-oxo-2-nonenal (ONE), pentenal, +14 carbonylation (+14 Ox) [31], oxidation (Ox), glutamic-semialdehyde (GSA), propanal, pentanal, heptanal, octanal, nonanal, tridecanal, tetradecanal, pentadecanal, octenal, and 5-oxo pentanoic acid.

Using previously-established procedures, the targeted analysis of glycated, AGE-modified, carbonylated/oxidized, and LMW carbonyl–protein adducts allowed the confident identification of 204 modified peptides (Table S2) corresponding to 189 modification sites in 108 milk proteins. Most modification sites were located on lysine (68.3%) and arginine residues (16.9%), less on threonine (4.8%), cysteine (3.2%), and histidine residues (2.6%), and only two or less on glutamine, leucine, glutamic acid, valine, and tyrosine residues (Figure 2a). Some lysine residues of abundant milk proteins were identified with different modification types indicating them as modification hotspots. For example, Lys32, Lys41, Lys158, and Lys199 of α_S2_-casein and lysine residues 91, 135, and 141 of β-lactoglobulin were glycated and modified by different AGEs (Table S3). Lys69 of β-lactoglobulin was detected as carbonylated and AGE-modified. Similarly, one residue in α-lactalbumin (Lys98), α_S1_-casein (Lys7), and β-casein (Lys176) was glycated and AGE-modified (Table S3). 

Around 45% of the modified peptides corresponded to abundant milk proteins, i.e., α_S1_-casein, α_S2_-casein, β-casein, κ-casein, β-lactoglobulin, α-lactalbumin, and serum albumin. Among them α_S2_-casein (30 peptides) and β-lactoglobulin (22 peptides) were the most common with ten out of 24 and twelve out of 15 lysine residues, respectively, being modified. 

Fourteen glycated proteins were identified by 51 lactosylated and 15 hexosylated peptides (Table S2, Figure 2b), which derived mostly from the major milk proteins with the highest numbers identified in α_S2_-casein (17 peptides) and β-lactoglobulin (16 peptides). Glycosylation-dependent cell adhesion molecule 1 (GLYCAM) carried three lactosylation sites identified by four peptides (Table S2). In the remaining six proteins one modification site was always identified, two proteins were lactosylated and four proteins were hexosylated (Tables S2 and S3).

Eighty-two AGE-modified peptides corresponding to 67 proteins were detected on lysine (76%) and arginine residues. The most common modifications were formyllysine (FL, 29 peptides), carboxymethylated lysine (CML, 16 peptides), and arginine (CMA, 10 peptides) accounting for ~70% of the identified AGEs (Figure 2b). Unlike Amadori products that were mostly detected in major milk proteins, AGEs were detected in a larger variety of proteins. Besides α_S2_-casein and β-lactoglobulin, which were both modified at six lysine or arginine residues, the other 65 proteins were modified only at one or two residues (Table S3). 

Thirty-six carbonylated/oxidized peptides from 26 proteins were identified (Table S2), i.e., nine HHE-derived peptides (6 × Lys, 2 × His, and 1 × Cys), seven peptides with oxidized threonine (T(Ox)), and six peptides with oxidized proline (P(GSA)) (Figure 2b). Around 42% of the peptides corresponded to the major milk proteins, i.e., peptides 3 to 6 from α-lactalbumin, peptides 54 to 57 from α_S2_-casein, and up to two peptides from α_S1_-casein, β-casein, κ-casein, β-lactoglobulin, and serum albumin (Table S2). Although fewer carbonylation/oxidation sites than glycation sites were identified, they were detected in more proteins.

### 3.2. LMW Carbonyl–Protein Adducts

Due to the high lipid content in raw milk, we assumed that aldehydes and ketones originating from oxidation of fatty acids and capable of reacting with proteins are present in raw milk and that processing may generate even more. The reactive carbonyls present in the processed flavored products were derivatized with CHH and identified by tandem mass spectrometry (Figure S1). Considering CHH-specific reporter ions, accurate masses, predicted elemental compositions, and compound-specific fragment ions [28], 21 reactive carbonyls were identified, i.e., eleven unsaturated alkanals (C_3_ to C_9_, C_11_, and C_13_ to C_15_), five hydroxylated alkanals, two alkenals, one oxo-carboxylic acid, and two dicarbonyls (Table S4). Consequently, 13 protein modifications presumably formed from the identified carbonyls were targeted in all samples (Table S1). Thus, 20 peptides derived from 15 proteins were identified (Table S2), 13 modified at lysine, six at arginine and only one at a histidine residue (peptide 162, Table S2). Among the major milk proteins, only α-lactalbumin was detected carrying this modification type (peptides 7 to 10, Table S2) including three different modifications formed by propanal, octenal, and 5-oxo pentanoic acid at Lys93 indicating that this site is prone to LMW carbonyl modifications. Additionally, the transport and Golgi organization protein 1 homolog was modified at Lys1239 and Lys1241 by octenal and Lys1460 by pentadecanal (peptides 194 to 196, Table S2). 

### 3.3. Influence of Processing and Flavoring

**Figure 3:**
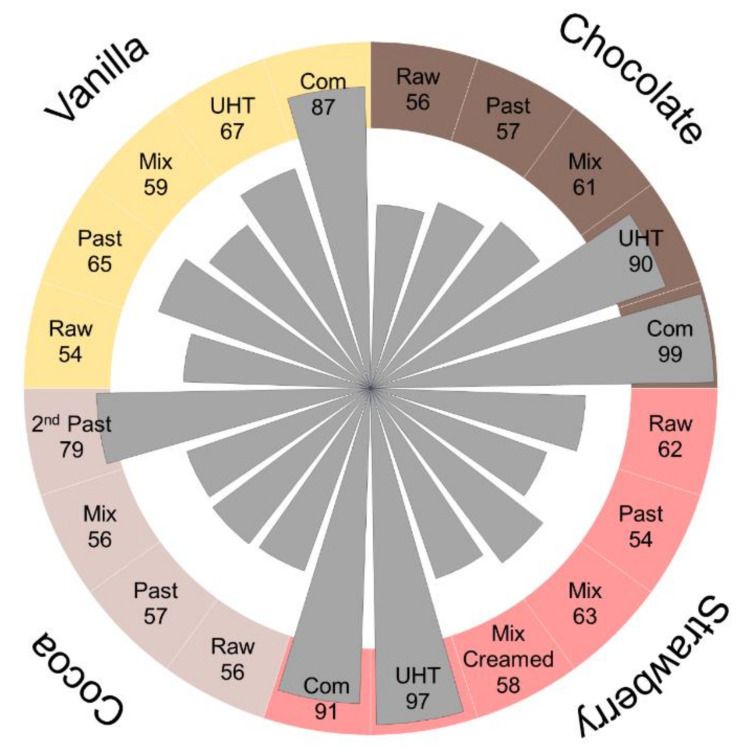
Overall numbers of modified peptides identified in each sample grouped by drinks and processing steps. Samples included raw milk (Raw), pasteurized milk (Past), milk mixed with flavorings (Mixed), milk drink after a second heat treatment, i.e., high-temperature-short-time (HTST, 2nd past) for cocoa and UHT for the other three drinks, and the corresponding milk drinks bought in a supermarket (Com).

**Figure 4:**
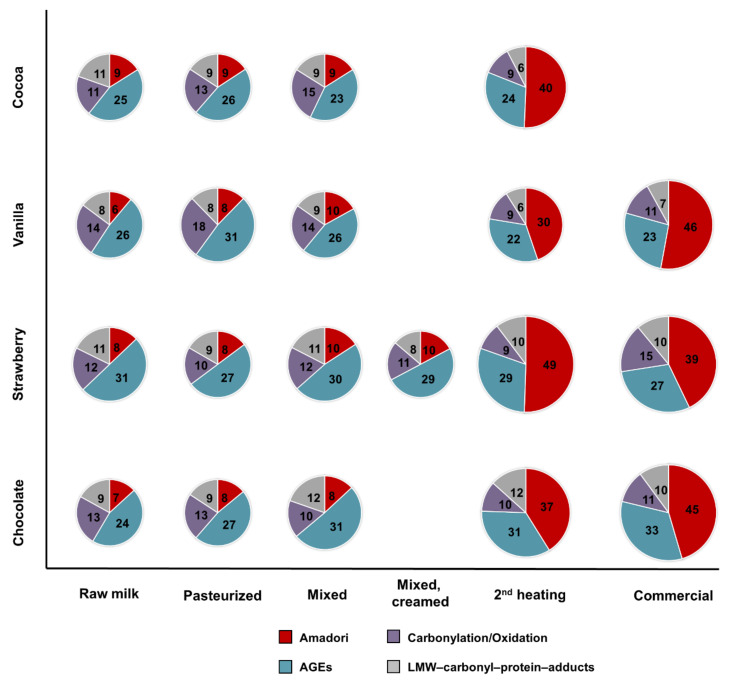
Contribution of each modification type to the overall number of identified modified peptides for the cocoa-, vanilla-, strawberry-, and chocolate-flavored milk drinks at each step of the processing chain. Samples included raw milk, pasteurized milk, milk mixed with flavorings, milk drink after the second heat treatment, and the commercial products. The size of the pie diagrams indicates the overall number of peptides.

The influence of processing and flavoring on protein modifications was studied for four flavored milk drinks by analyzing samples collected along the processing chains (Figure 3 and Figure 4). As expected, similar numbers of modified peptides (i.e., 54 to 62 peptides) were detected in the tryptic digests of all samples taken from four different raw milk batches (Figure 3). Specifically, only six to nine Amadori peptides (Figure 4, raw milk), eleven to fourteen carbonylation/oxidation modifications, eight to eleven LMW carbonyl-protein adducts, and 24 to 31 AGEs were detected (Figure 4). 

The first two samples taken after the initial pasteurization and mixing step (addition of flavorings and further ingredients) showed no significant effect on the peptide numbers of each modification type (Figure 3 and Figure 4, pasteurized and mixed). It should be noted that a peptide was considered identified in a sample when confirmed by one tandem mass spectrum acquired in the targeted MS strategy. The slightly lower numbers of peptides in the pasteurized samples indicate that a few peptides were not confidently identified, but their presence was confirmed by the corresponding MS signals at the expected retention times. Importantly, the slight variations in the numbers of peptides identified in the samples collected in the initial processing steps were comparable to variations seen among the four different raw milk samples.

The second thermal treatment affected the numbers of modified peptides in all samples, mostly the numbers of Amadori peptides increasing 4.5 to sixfold relative to the initial raw milk samples. The highest numbers of 90 and 97 modified peptides were detected in UHT chocolate and UHT strawberry drinks, respectively, and the lowest numbers in pasteurized cocoa (79) and surprisingly in UHT vanilla (67) drinks (Figure 4, 2nd heating). In strawberry UHT milk, Amadori peptides represented 50.5% of all identified modified peptides. The increase in identified Amadori peptides was slightly lower for the other UHT milk drinks with glycated peptides accounting for 41% of all modified peptides in chocolate UHT and 45% in vanilla UHT milk. Noteworthy, cocoa milk treated by pasteurization as a second heat processing, showed a comparable increase in the number of Amadori peptides as in the UHT drinks and this modification type represented 51% of all identified modifications (Figure 4, 2nd heating). The numbers of AGE-modified, carbonylated/oxidized, and LMW carbonyl-modified peptides did not change significantly and were thus similar to the numbers in the raw milk samples (Figure 4, 2nd heating). 

The numbers of detectable Amadori peptides changed in the packed and commercialized UHT drinks of the same batch bought a few weeks later in a supermarket (Figure 3 and Figure 4, Commercial), while the numbers of AGEs, carbonylated/oxidized peptides, and peptides modified by LMW carbonyls were mostly unaffected. The number of Amadori peptides increased in the vanilla and chocolate milk drinks from 30 to 46 and 37 to 45, respectively, but decreased by ten peptides in the strawberry milk drink, which in turn showed an increase of six carbonylated/oxidized peptides. 

Relative quantitation of several modified peptides from the major milk proteins underlined the trend observed for Amadori peptides. The contents were not affected by the initial processing steps and only increased in the final heating step, as shown for peptides 26 and 43 (Figure S2). Furthermore, the contents of the few quantifiable FL peptides, as the most abundant AGE modification detected, were relatively stable along the whole production chain for all drinks, as can be nicely seen for peptide 74 (Figure S2). On the other hand, pyrraline might be more sensitive to harsher processing, as it was only quantifiable in samples taken from the final and commercial products (peptide 63, Figure S2). However, these are preliminary assumptions, as we aimed at the simultaneous detection of a large number of different PTMs without applying modification-specific enrichment strategies, which are a prerequisite for higher signal intensities allowing a reliable quantitation, ideally by using internal standards.

## 4. Discussion

Industrial production of milk drinks alters the composition of milk due to the addition of flavorings and other ingredients as well as thermal treatments. This may enhance Maillard reactions and oxidations yielding Amadori products and oxidized or AGE-/ALE-modified proteins. These reactions have already occurred in raw milk at low levels defining basic modification degrees of milk proteins. The enhancement of Maillard reactions and protein carbonylation by thermal processing is well documented for bovine milk [10,17,18,19,20,21,32,33]. For example, the number of lactosylation sites and lactosylation degrees increase significantly from raw to pasteurized milk and further to UHT milk, whereas the number of AGE-modified residues are similar in raw and pasteurized milk, but increase during UHT treatment [19,20,21,33]. In the same way, the levels of furosine and CML content increases from pasteurized to UHT milk [34]. Pasteurization and UHT treatment also increase protein-bound carbonyls [22,23]. Despite the great demand for flavored milk products, it has not been investigated whether flavorings or other ingredients and especially the two thermal treatments affect the bovine milk proteome during the manufacturing process. To study differences in protein modifications more precisely, samples should be analyzed from the initial raw milk along all major processing steps until the final product for a single batch. In order to evaluate the influence of the processing chain and the addition of further ingredients such as flavorings on protein modifications, we studied samples collected for cocoa-, chocolate-, strawberry-, and vanilla-flavored milk drinks. To the best of our knowledge, this is the most comprehensive proteomics study on bovine milk targeting 39 different PTMs including modifications resulting from 13 LMW carbonyls identified in the organic phases that have not been targeted as PTMs before. 

Among the identified PTMs, lactosylation and formylation of lysine residues dominated, followed by carboxymethylation and hexosylation, confirming previous results [20,21]. Considering the protein targets of glycation and AGE formation, it seems that early glycation reactions mainly affect the major milk proteins, whereas AGEs are formed at various proteins, but obviously at the detected sites at rather high levels considering the low abundances of these proteins. Interestingly, this study confirms recent reports indicating that α_S2_-CN and β-LG are preferential targets of glycation [19,20,21], whereas α_S2_-CN is the main carbonylation target followed by α-LA [35]. Oxidation/carbonylation sites and adducts with LMW carbonyls appear to be less common. 

The four raw milk batches were very similar in terms of numbers and distributions of identified modification types (basic modification degrees) and neither the first thermal treatment (pasteurization) nor the addition and mixing with flavorings affected them significantly. However, the second thermal treatment, i.e., HTST for cocoa and UHT for the other drinks, increased the number of lactosylation sites considerably shifting the distribution towards Amadori peptides. Judging the relative quantities of several Amadori peptides by the relative peak areas revealed higher contents after the final processing step and in the commercial products, confirming the observed changes in the numbers of identified peptides, which is in agreement with a previous report on regular milk [35]. While processing increased the number of Amadori products, new AGEs and protein-bound carbonyls were most likely not formed or were at very low levels, below the limits of detection. Noteworthy, fewer AGE-modifications were identified in the flavored milk drinks than reported for conventional milk [21]. However, the numbers reported in pasteurized and UHT products varied among different brands indicating that the processing conditions applied by different companies may determine the final modification degrees [21]. Additionally, AGEs might be affected in different ways, as relative quantitation of FL- and pyrraline-modified peptides indicated that FL levels are mostly independent of thermal treatment, whereas pyrraline appeared only after the second heating step. The relatively stable quantities of FL-modified peptides after pasteurization and UHT are in a good agreement with previous studies on AGEs in regular and hay milk [21,35]. However, the effects of pasteurization and UHT on AGEs probably depend on the exact processing conditions, as they differ among brands [21]. Although the numbers of carbonylated peptides identified in the flavored milk drinks are rather low, they are comparable to those reported for raw and processed milk samples [22]. The increase of carbonylated peptides reported by this study for UHT products derived mainly from the relatively-unstable glyoxal–protein adducts, which can be detected after derivatization and enrichment. As this strategy was not applied here, the isobaric glyoxal and CML modifications are reported here as CML assuming that the glyoxal–protein adducts containing a reactive carbonyl group were mostly missed [22]. Nevertheless, the numbers of identified carbonylated peptides were still quite low compared to Amadori and AGEs and therefore it is difficult to judge their changes among the samples. Importantly, the group of carbonylation/oxidation modifications also includes LPP adducts, such as those formed from HHE and ONE, resulting from lipid peroxidation.

Modifications from LMW carbonylated-lipids and other small aldehydes that do not produce reactive carbonyls in proteins were grouped as LMW carbonyl–protein adducts. Prior to targeted analysis, the final products of each drink were derivatized with CHH and the reactive carbonyl compounds were manually identified considering exact masses and specific fragment ions (Figure S1). Thus, 21 LMW carbonyl-derived compounds, which included C_3_ to C_9_, C_11_, and C_13_ to C_15_ aldehydes, being in a good agreement with studies reporting free volatile compounds in milk, were confirmed [15,24,25,36]. Analysis of carbonylated compounds present in the fat phase of spontaneously-oxidized whole milk identified saturated C_5_ to C_16_ aldehydes [36]. The authors assumed that saturated C_11_ to C_16_ aldehydes result from lipid synthesis or hydrolysis during pasteurization and not from autoxidation [36]. Furthermore, C_5_ to C_10_ aldehydes were identified at similar levels in raw and pasteurized milk, but at higher concentrations in UHT milk e.g., heptanal and decanal [15,24]. Similarly, higher concentrations of volatile species were found in UHT milk containing 3% than in UHT milk containing 1% fat, e.g., for hexanal [15]. The procedure used here to identify small reactive carbonyls in flavored milk relied on CHH-labeling followed by high resolution MS, which was developed on standard fatty acid mixtures [28]. However, CHH-derivatization is applicable to food systems as well. Recently, a similar CHH-labeling procedure was adapted to mayonnaise, allowing the identification and semi-quantitation of volatile aldehydes [37]. Similarly, CHH-derivatization identified docosahexanoic acid oxidative markers in milk powders [38] indicating its adaptability to food matrices. Although free reactive carbonyls were analyzed in milk before, studies focusing on the reaction products formed with milk proteins are still lacking. The numbers of identified PTMs resulting from the identified small carbonylated lipid species were similar in flavored milk drinks with different fat contents. In general, the numbers of this type of modified peptide and deviations among samples were relatively low, indicating that these reactions are not very pronounced in bovine milk.

## 5. Conclusions

This is the first study targeting 26 different PTMs related to glycation, AGE-formation, carbonylation, and lipid oxidation and additionally 13 sample-specific LMW carbonyl adducts in the bovine milk proteome. The study will provide a better understanding of the complex reactions occurring during the production of milk drinks flavored with cocoa, strawberry, chocolate, and vanilla. The relatively low numbers and levels of the targeted modification types were not affected by the initial pasteurization step and mixing with flavorings. However, the second heating step considerably increased, in particular, lactosylation degrees in all drinks, irrespective of flavoring and fat content. Thus, the final products contained high number and relative quantities of Amadori peptides, while the numbers of AGEs, oxidized/carbonylated peptides, and adducts with LMW carbonyls remained similar. However, the influence on the studied modification types might vary, as exemplified for rather stable FL levels but increasing pyrraline concentrations only quantifiable in the final products. Lactosylation, formylation, and carboxymethylation were the main non-enzymatic protein modifications identified in bovine milk, but only lactosylation increases after thermal processing.

## Supplementary Materials

The following are available online at https://www.mdpi.com/2076-3921/9/11/1169/s1, Figure S1: Tandem mass spectrum of a CHH-derivatized carbonyl, Figure S2: Relative quantities of Amadori- and AGE-modified peptides among all samples, Table S1: List of targeted dynamic modification, Table S2: Modified peptides identified in any of the analyzed milk samples, Table S3: Modification sites sorted by proteins and types, Table S4: Overview of reactive carbonyl species identified using CHH-derivatization.
