# Supplementary material for: Profiling of Low-Molecular-Weight Carbonyls and Protein Modifications in Flavored Milk
Source: Antioxidants (Basel). 2020 Nov 23;9(11):1169. doi: 10.3390/antiox9111169 (PMC7700654; doi:10.3390/antiox9111169)
Supplement: Supplementary file 1 [file antioxidants-09-01169-s001.pdf]

Supplementary material

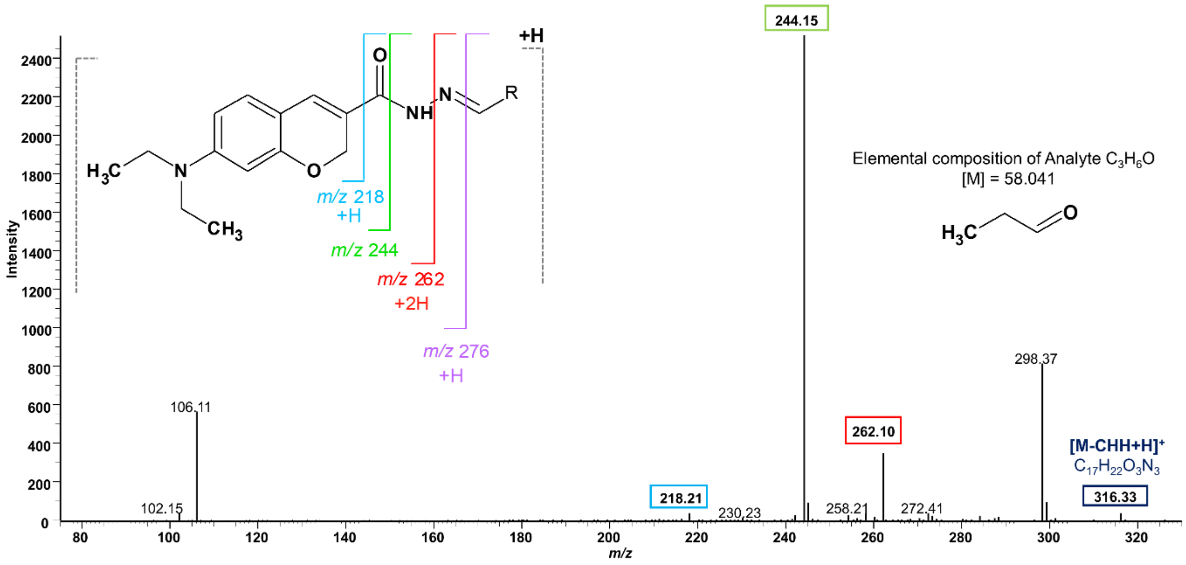

Figure 1. CID tandem mass spectrum of CHH-derivatized propanal (*m/z* 316.33) acquired by gas-phase fractionation on LTQ Orbitrap XL mass spectrometer. Tandem mass spectra were manually annotated if characteristic CHH-reporter ions were present.

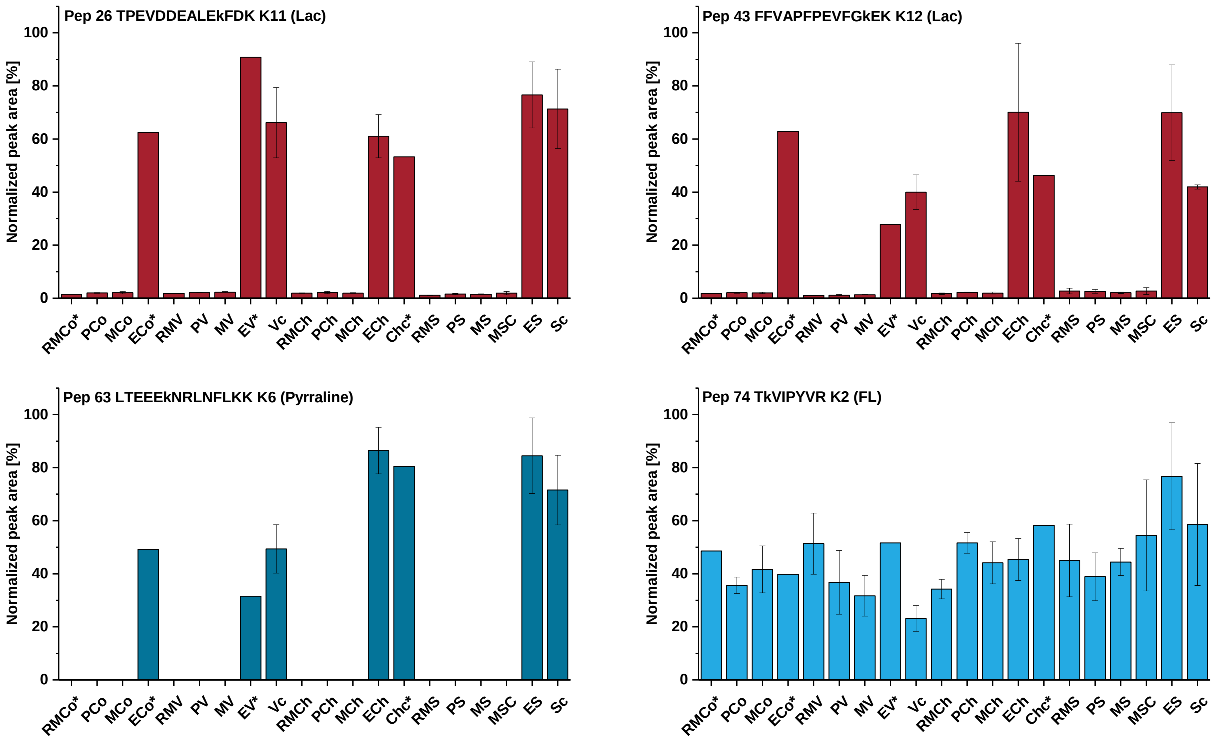

Figure 2. Relative peptide quantities presented as normalized peak areas (%). Ratios were calculated by dividing the peak are of the modified peptide to the sum of unmodified peptides of the same protein (seven peptides for β-lactoglobulin, six peptides for α_{S1}-casein, and eleven peptides for α_{S2}-casein) and normalized to the highest observed ratio (%). Peptide 27 is lactosylated at Lys135 of β-lactoglobulin. Peptide 44 is lactosylated at Lys34 of α_{S1}-casein. Peptides 64 and 76 correspond to α_{S2}-casein carrying pyrraline and formyllysine at Lys158 and Lys199, respectively. An asterisk (*)

indicates that only two replicates were acquired for this sample. Abbreviations for milk drinks: Ch. for chocolate milk drink, S for strawberry milk drink, V for vanilla milk drink, and Co for cocoa milk drink. Collected samples are labeled as follows: RM indicates raw milk, P pasteurization, M mixing, MSC stands for the mixed strawberry milk with an additional creaming step, E stands for the end product after a second heating step (HTST for Co, and UHT for Ch, S, V), and c denotes the milk drink obtained from a supermarket.

Table 1. Overview of targeted modifications. Modifications corresponding to early glycation are highlighted in red, AGEs in blue, carbonylation/oxidation in violet, and LMW carbonyl-protein adducts in grey. Modifications are grouped by templates used for data processing. Each template additionally contained carbamidomethylation (Cam, +57.02 Da) of cysteine and methionine oxidation (Ox, +15.995 Da).

Modification	Abbreviation	Mass Shift [Da]*	Amino Acid Residue
hexosylation	Hex	+162.053	K
lactosylation	Lac	+324.106	K
carboxyethyl	CEL/CEA	+72.021	K, R
carboxymethyl	CML/CMA	+58.005	K, R
glyoxal-derived hydroimidazolium	Glarg	+39.995	R
methylglyoxal-derived hydroimidazolones	MGH	+54.011	R
argpyrimidine	Argpyr	+80.026	R
glyceraldehyde-derived pyridinium	GLAP	+109.029	K
tetrahydropyrimidine	THP	+144.042	R
pyrraline		+108.021	K
formylation	FL	+27.995	K
acetylation	AL	+42.011	K
glycerinyl lysine	GL	+88.016	K
oxalic acid monolysinylamide	OMA	+71.985	K
oxidation	Ox	+15.995	P (glutamic-semialdehyde, GSA), C, Y
oxidation	Ox	-2.016	T (2-amino-3-ketobutyric acid)
Glutamic-semialdehyde	GSA	-43.053	R
2-amino-adipic-semialdehyde	AAS	-1.032	K
+14 Da carbonylation [1]	+14	+13.979	A, E, I, K, L, Q, V
acrolein	ACR	+56.026	C, H, K
crotonaldehyde	CRO	+70.042	C, H, K
pentenal	Pent	+84.060	C, H, K
4-hydroxy-2-hexenal	HHE	+114.068	C, H, K
4-hydroxy-2-nonenal	HNE	+156.115	C, H, K
4-oxo-2-hexenal	OHE	+112.052	C, H, K
4-oxo-2-nonenal	ONE	+154.099	C, H, K
propanal		+40.031	K, R
pentanal		+68.063	K, R
hexanal		+82.078	K, R
heptanal		+96.094	K, R
octenal		+126.104	C, H, K
nonenal		+140.120	C, H, K
5-oxo pentanoic acid		+98.099	K, R

octanal	+110.109	K, R
nonanal	+124.125	K, R
undecanal	+152.156	K, R
pentadecanal	+208.219	K, R
tetradecanal	+194.204	K, R
tridecanal	+180.188	K, R

*The indicated mass shifts do not confirm the structure of the proposed modifications and it cannot be excluded that they match to other modifications not considered in this study.

Table 2. Modified peptides identified in any of the analyzed milk samples. The Xcorr-values represent the highest value obtained for a peptide in any analysis. Protein modification sites are numbered for the mature protein without pro- or signal-peptide sequences as indicated in UniProtKB.

Nr.	Accession	Description	Mod. Site Protein	Sequence	Mod. Site Peptide [a)]	z	*m*/*z*	t_R [min]	Xcorr
1	P00711	Alpha lactalbumin	K5	EQLTkCEVFR	K5(Lac)	3	526.25	27.37	2.2
2	P00711	Alpha lactalbumin	K5	EQLTkcEVFR	K5(Lac); C6(Cam)	3	545.26	23.31	2.6
3	P00711	Alpha-lactalbumin	T86	DDQNPHSSNICNISCDKFLDDDLtDDIMCVKK	T24(Ox)	5	728.72	51.34	3.7
4	P00711	Alpha-lactalbumin	T86	IWCKDDQNPHSSNICNISCDKFLDDDLtDDIMcVKK	T28(Ox); C33(Cam)	6	705.32	49.83	3.5
5	P00711	Alpha-lactalbumin	T86	IWCKDDQNPHSSNICNIScDKFLDDDLtDDIMcVKK	C19(Cam); T28(Ox); C33(Cam)	6	714.82	50.41	4.7
6	P00711	Alpha-lactalbumin	T86	IWcKDDQNPHSSNICNISCDKFLDDDLtDDIMcVKK	C3(Cam); T28(Ox); C33(Cam)	6	714.82	48.96	3.9
7	P00711	Alpha-lactalbumin	K93	IWCKDDQNPHSSNICNISCDKFLDDDLTDDImCVkK	M32(Ox); K35(5-oxo pentanoic acid)	5	857.99	49.62	4.2
8	P00711	Alpha-lactalbumin	K93	IWCKDDQNPHSSNIcNISCDKFLDDDLTDDIMcVk	C15(Cam); C33(Cam); K35(Octenal)	5	857.58	50.38	4.5
9	P00711	Alpha-lactalbumin	K93	IWcKDDQNPHSSNICNISCDKFLDDDLTDDIMcVk	C3(Cam); C33(Cam); K35(Octenal)	5	857.58	48.93	4.6
10	P00711	Alpha-lactalbumin	K93	IWCKDDQNPHSSNICNIScDKFLDDDLTDDImCVkK	C19(Cam); M32(Ox); K35(Propanal)	6	714.99	50.52	5.2
11	P00711	Alpha lactalbumin	K98	ILDkVGINYWLAHK	K4(Lac)	3	665.35	42.54	2.8
12	P00711	Alpha lactalbumin	K98	ILDkVGINYWLAHK	K4(FL)	3	566.65	56.36	2.6
13	P00711	Alpha lactalbumin	K98	KILDkVGINYWLAHK	K5(Lac)	3	708.05	40.59	3.1
14	P02754	Beta lactoglobulin	K47	VYVEELkPTPEGDLEILLQK	K7(Lac)	3	879.79	52.23	3.3
15	P02754	Beta lactoglobulin	K47	VYVEELkPTPEGDLEILLQKWENGEcAQKK	K7(Lac); C26(Cam)	5	774.39	57.23	3.4
16	P02754	Beta lactoglobulin	K69, K70	VYVEQLKPTPEGDLEILLQKWENGECAQkk	K29(GL); K30(FL)	5	721.17	59.00	4.2
17	P02754	Beta lactoglobulin	C66, K69	VYVEQLKPTPEGDLEILLQKWENGEcAQk	C26(Ox); K29(HHE)	5	698.36	58.10	3.9
18	P02754	Beta lactoglobulin	K75	IIAEkTKIPAVFKIDALNENK	K5(Lac)	4	670.62	40.43	5.3
19	P02754	Beta lactoglobulin	K77	TkIPAVFKIDALNENK	K2(Lac)	3	709.05	39.49	3.1
20	P02754	Beta lactoglobulin	K77	TkIPAVFKIDALNENK	K2(FL)	3	610.34	46.29	3.3
21	P02754	Beta lactoglobulin	K83	TKIPAVFkIDALNENK	K8(Lac)	3	709.05	39.54	3.2
22	P02754	Beta lactoglobulin	K91	IDALNENkVLVLDTDYKK	K8(Lac)	4	604.56	37.46	3.9
23	P02754	Beta lactoglobulin	K91	IDALNENkVLVLDTDYKK	K8(FL)	3	707.05	47.00	4.2
**Nr.**	**Accession**	**Description**	**Mod. site protein**	**Sequence**	**Mod. site peptide [a)]**	**z**	***m*/*z***	**t_R [min]**	**Xcorr**
24	P02754	Beta lactoglobulin	K100, K101	IDALNENKVLVLDTDYkk	K17(Hex); K18(Hex)	4	604.57	37.59	4.1
25	P02754	Beta lactoglobulin	K135	TPEVDDEALEkFDK	K11(Hex)	3	599.95	33.85	3.9
26	P02754	Beta lactoglobulin	K135	TPEVDDEALEkFDK	K11(Lac)	3	653.97	33.70	3.4
27	P02754	Beta lactoglobulin	K135	TPEVDDEALEkFDKALK	K11(Lac)	4	568.78	40.82	5.4
28	P02754	Beta lactoglobulin	K135	TPEVDDEALEkFDKALK	K11(FL)	3	659.33	47.45	2.2

29	P02754	Beta lactoglobulin	K135	TPEVDDEALEkFDKALKALPMHIR	K11(Lac)	5	618.92	53.54	4.3
30	P02754	Beta lactoglobulin	K138	TPEVDDEALEKFDkALK	K14(Hex)	3	704.02	41.30	3.2
31	P02754	Beta lactoglobulin	K138	TPEVDDEALEKFDkALK	K14(Lac)	4	568.78	41.30	5.0
32	P02754	Beta lactoglobulin	K141	ALkALPMHIR	K3(Hex)	3	437.92	26.29	2.2
33	P02754	Beta lactoglobulin	K141	ALkALPMHIR	K3(Lac)	3	491.94	26.28	2.6
34	P02754	Beta lactoglobulin	K141	ALkALPMHIR	K3(Pyrraline)	3	419.91	26.10	3.4
35	P02754	Beta lactoglobulin	K141	TPEVDDEALEKFDKALk	K17(Lac)	4	568.78	41.43	4.8
36	P02662	Alpha-S1-casein	P5	HpIKHQGLPQEVLNENLLR	P2(GSA)	3	751.08	40.22	2.7
37	P02662	Alpha-S1-casein	K7	HPIkHQGLPQEVLNENLLR	K4(Lac)	4	640.59	36.94	3.8
38	P02662	Alpha-S1-casein	K7	HPIkHQGLPQEVLNENLLR	K4(FL)	4	566.56	40.65	5.0
39	P02662	Alpha-S1-casein	P29	FFVAPFpEVFGK	P7(GSA)	2	700.87	56.30	1.8
40	P02662	Alpha-S1-casein	K34	FFVAPFPEVFGkEK	K12(Hex)	3	601.98	52.69	1.8
41	P02662	Alpha-S1-casein	K34	FFVAPFPEVFGkEKVNELSK	K12(Hex)	4	619.33	51.42	2.9
42	P02662	Alpha-S1-casein	K34, K36	FFVAPFPEVFGkEkVNELSK	K12(Hex); K14(Hex)	4	659.84	51.30	3.9
43	P02662	Alpha-S1-casein	K34	FFVAPFPEVFGkEK	K12(Lac)	3	656.00	52.46	2.9
44	P02662	Alpha-S1-casein	K34	FFVAPFPEVFGkEKVNELSK	K12(Lac)	4	659.84	51.11	4.5
45	P02662	Alpha-S1-casein	K36	FFVAPFPEVFGKEkVNELSK	K14(Lac)	4	659.84	51.10	4.1
46	P02662	Alpha-S1-casein	K132	EGIHAQQkEPMIGVNQELAYFYPELFR	K8(Lac)	4	883.68	59.50	3.1
47	P02663	Alpha-S2-casein	K32	NMAINPSkENLcSTFcK	K8(FL); C12(Cam); C16(Cam)	3	681.31	35.87	2.0
48	P02663	Alpha-S2-casein	K32	NMAINPSkENLCSTFCK	K8(CML)	3	653.30	40.07	2.0
49	P02663	Alpha-S2-casein	K32	NMAINPSkENLCSTFcK	K8(CML); C16(Cam)	3	672.31	37.49	2.4
50	P02663	Alpha-S2-casein	K32	NMAINPSkENLcSTFCK	K8(Lac); C12(Cam)	3	761.01	36.43	1.9
**Nr.**	**Accession**	**Description**	**Mod. site protein**	**Sequence**	**Mod. site peptide [a)]**	**z**	***m/z***	**t_R [min]**	**Xcorr**
51	P02663	Alpha-S2-casein	K32	NMAINPSkENLCSTFCK	K8(Lac)	3	742.00	39.18	1.9
52	P02663	Alpha-S2-casein	K32	NMAINPSkENLCSTFcK	K8(Lac); C16(Cam)	3	761.01	36.83	2.2
53	P02663	Alpha-S2-casein	K32	NMAINPSkENLcSTFcK	K8(Lac); C12(Cam); C16(Cam)	3	780.01	34.40	2.4
54	P02663	Alpha-S2-casein	T38	NMAINPSKENLCStFCK	T14(Ox)	3	633.29	71.94	2.1
55	P02663	Alpha-S2-casein	T38	NMAINPSKENLCStFCKEVVR	T14(Ox)	4	596.04	42.29	3.9
56	P02663	Alpha-S2-casein	C36, C40	NMAINPSKENLcSTFcKEVVR	C12(Pent); C16(Ox)	4	621.55	39.57	3.1
57	P02663	Alpha-S2-casein	C40, K41	NMAINPSKENLCSTFckEVVR	C16(Ox); K17(Pent)	4	621.55	39.46	3.1
58	P02663	Alpha-S2-casein	K41	NMAINPSKENLcSTFCk	C12(Cam); K17(CML)	3	672.31	37.29	3.3
59	P02663	Alpha-S2-casein	K41	NMAINPSKENLcSTFCkEVVR	C12(Cam); K17(CML)	4	625.30	40.75	3.4
60	P02663	Alpha-S2-casein	K41	NMAINPSKENLCSTFCkEVVR	K17(Lac)	4	677.57	45.17	2.8
61	P02663	Alpha-S2-casein	K41	NMAINPSKENLcSTFCkEVVR	C12(Cam); K17(Lac)	4	691.83	41.02	2.8
62	P02663	Alpha-S2-casein	K152	TkLTEEEKNRLNFLK	K2(FL)	4	473.51	35.87	3.2
63	P02663	Alpha-S2-casein	K158	LTEEEkNRLNFLKK	K6(Pyrraline)	3	624.01	25.89	1.7
64	P02663	Alpha-S2-casein	K158	LTEEEkNRLNFLK	K6(Lac)	4	490.25	30.09	4.0
65	P02663	Alpha-S2-casein	K158	LTEEEkNRLNFLKK	K6(Hex)	4	481.76	26.15	4.1
66	P02663	Alpha-S2-casein	K158	LTEEEkNRLNFLKK	K6(Lac)	4	522.28	26.31	5.2

67	P02663	Alpha-S2-casein	K158	TKLTEEEkNRLNFLK	K8(Lac)	4	547.54	30.01	5.2
68	P02663	Alpha-S2-casein	K165	LTEEEKNRLNFLkK	K13(Lac)	4	522.28	26.14	3.6
69	P02663	Alpha-S2-casein	K173	YQkFALPQYLK	K3(Lac)	3	574.97	39.26	2.9
70	P02663	Alpha-S2-casein	K181	FALPQYLkTVYQHQK	K8(Lac)	4	547.78	42.67	4.8
71	P02663	Alpha-S2-casein	K188	TVYQHQkAMKPWIQPK	K7(Lac)	5	462.24	23.07	3.7
72	P02663	Alpha-S2-casein	K191	AMkPWIQPK	K3(Hex)	3	420.89	23.71	1.9
73	P02663	Alpha-S2-casein	K191	AMkPWIQPK	K3(Lac)	3	474.91	23.86	2.5
74	P02663	Alpha-S2-casein	K199	TkVIPYVR	K2(FL)	2	502.30	31.40	1.4
75	P02663	Alpha-S2-casein	K199	TkVIPYVR	K2(Lac)	3	433.91	21.76	1.4
76	P02663	Alpha-S2-casein	R205	VIPYVrYL	R6(MGH)	2	538.81	46.20	1.8
77	P02666	Beta-casein	K176	VLPVPQkAVPYPQR	K7(Hex)	3	585.33	30.63	2.4
78	P02666	Beta-casein	K176	VLPVPQkAVPYPQR	K7(Lac)	3	639.35	30.45	2.3
**Nr.**	**Accession**	**Description**	**Mod. Site Protein**	**Sequence**	**Mod. Site Peptide** [a)]	**z**	***m/z***	**t_R [min]**	**Xcorr**
79	P02666	Beta-casein	K176	VLPVPQkAVPYPQR	K7(CML)	3	550.65	33.66	2.2
80	P02666	Beta-casein	P186	DMpIQAFLLYQEPVLGPVR	P3(GSA)	3	734.73	61.44	2.5
81	P02668	Kappa-casein	K21	FFSDkIAK	K5(Lac)	3	427.22	21.75	2.3
82	P02668	Kappa-casein	K24	FFSDKIAkYIPIQYVLSR	K8(Lac)	4	628.84	56.04	4.0
83	P02668	Kappa-casein	K24	IAkYIPIQYVLSR	K3(Lac)	3	630.02	44.86	2.7
84	P02668	Kappa-casein	K46	YPSYGLNYYQQkPVALINNQFLPYPYYAKPAAVR	K12(Lac)	5	867.64	54.34	7.1
85	P02668	Kappa-casein	K46, K63	YPSYGLNYYQQkPVALINNQFLPYPYYAkPAAVR	K12(Hex); K29(Hex)	4	1084.30	54.41	2.8
86	P02668	Kappa-casein	K86	SPAQILQWQVLSNTVPAkScQAQPTTMAR	K18(Lac); C20(Cam)	4	884.69	48.56	2.2
87	P02668	Kappa-casein	K86	SPAQILQWQVLSNTVPAkSCQAQPTTMAR	K18(Lac)	4	870.44	49.93	2.7
88	P02668	Kappa-casein	P99	HpHPHLSFMAIPPK	P2(GSA)	3	542.29	35.92	2.0
89	P02668	Kappa-casein	P101	HPHpHLSFMAIPPK	P4(GSA)	3	542.29	35.90	1.9
90	P02769	Serum albumin	C476	CcTESLVNRRPcFSALTPDETYVPK	C2(HHE); C12(Cam)	4	750.86	40.71	3.4
91	P02769	Serum albumin	K232	AEFVEVTkLVTDLTK	K8(Lac)	3	673.02	56.12	2.3
92	P02769	Serum albumin	K275	LkECCDKPLLEK	K2(Lac)	3	581.62	20.92	1.9
93	P80195	Glycosylation-dependent cell adhesion molecule 1	K71	LPLSILkEK	K7(Lac)	3	455.60	33.21	2.0
94	P80195	Glycosylation-dependent cell adhesion molecule 1	K71	QPQSQNPKLPLSILkEK	K15(Lac)	4	568.81	34.75	3.0
95	P80195	Glycosylation-dependent cell adhesion molecule 1	K73	LPLSILKEk	K9(Lac)	3	455.60	33.09	2.4
96	P80195	Glycosylation-dependent cell adhesion molecule 1	R109	ImrNLENTVKETIK	M2(Ox); R3(Heptanal)	3	601.01	35.48	2.3
97	P80195	Glycosylation-dependent cell adhesion molecule 1	K116	NLENTVkETIKYLK	K7(Lac)	3	673.02	45.32	2.1
98	Q2KJ46	26S proteasome non-ATPase regulatory subunit 3	K503	FPPKSYNkDLESAEER	K8(AL)	3	651.32	72.66	2.4
99	Q3T0X6	40S ribosomal protein S16	K33, R37	RGNGLIkVNGrPLEMIEPR	K7(CML); R11(MGH)	4	566.06	40.30	2.8

Nr.	Accession	Description	Mod. Site Protein	Sequence	Mod. Site Peptide [a)]	z	*m/z*	tR [min]	Xcorr
100	Q58DW5	60S ribosomal protein L5	K241, K242	QFSQYIKNNVTPDMMEEMYkk	K20(FL); K21(FL)	4	670.81	29.73	2.0
101	Q9GLE5	72 kDa type IV collagenase	K42	EScNLFVLkDTLKK	C3(Cam); K9(Pentanal)	3	588.33	25.67	2.6
102	Q9TT92	A disintegrin and metalloproteinase with thrombospondin motifs 5	R144	GrICLQGKcVDK	R2(THP); C9(Cam)	3	507.59	25.96	2.7
103	A4IFD0	Adenylate kinase isoenzyme 5	K389	KcKIIFMIGGPGSGkGTQCGK	C2(Cam); K15(FL)	3	732.38	40.88	2.1
104	Q3SZF2	ADP-ribosylation factor 4	R19	QmrILmVGLDAAGK	M2(Ox); R3(Heptanal); M6(Ox)	3	544.31	43.92	2.5
105	P20000	Aldehyde dehydrogenase, mitochondrial	R89, R96	LLNrLADLIErDR	R4(CMA); R11(CMA)	3	571.65	49.04	2.1
106	Q3MHM0*	Amyloid beta (A4) protein-binding, family B, member 1 interacting protein	K282, K291	KENkETNEKmNAk	K4(FL); M10(Ox); K13(FL)	3	545.93	88.44	2.1
107	Q3SWX7	Annexin A3	K169, K177	RDESLkVDEQLARk	K6(FL); K14(FL)	3	581.64	42.60	1.9
108	Q7YRZ2	Aprataxin	K61, K62	IADkkCSRQQVQLK	K4(FL); K5(OMA)	3	582.30	50.77	2.7
109	Q29RI0	Atypical kinase COQ8A, mitochondrial	K612	LVPPPEETYSLHRk	K14(FL)	3	565.30	43.65	1.9
110	Q5I597	Betaine--homocysteine S-methyltransferase 1	L145, V149	KVFQQQlEVFvKK	L7(+14 Ox); V11(+14 Ox)	3	550.31	28.23	2.3
111	Q5I597	Betaine--homocysteine S-methyltransferase 1	Q142, V149	KVFqQQLEVFvKK	Q4(+14 Ox); V11(+14 Ox)	3	550.31	27.60	2.3
112	Q32S26	Bromodomain-containing protein 2	K329, K342	kDLPDSQQQHQSSkK	K1(OMA); K14(OMA)	3	633.29	88.83	2.0
113	Q3SWX5	Cadherin-6	R684	rTPAARDNTDVRDFINQR	R1(Argpyr)	3	742.38	43.52	2.4
114	Q29RI6	Calcium-binding protein 39	H153	hEPLAK	H1(HHE)	2	404.73	27.04	1.1
115	Q58CW6	Calcium-responsive transcription factor	K347	mEQEkAFNLLKK	M1(Ox); K5(ONE)	3	550.31	28.16	2.1
116	Q08DY9	Caspase-3	K197, R201	kHAHrLELMHILTRVNR	K1(CEL); R5(Glarg)	4	559.81	36.08	3.0
117	Q29RT4	Cell division cycle-associated protein 2	R821	YrGHSVGGSDGPGLHLER	R2(CMA/CML)	3	651.32	41.71	2.6
Nr.	Accession	Description	Mod. site protein	Sequence	Mod. site peptide [a)]	z	*m/z*	tR [min]	Xcorr
118	Q32LN4*	Coiled-coil domain containing 19	K390	AQDYQAEQDALRAk	K14(Hex)	3	590.29	30.98	2.1
119	A0JNH6	Coiled-coil domain-containing protein 102A	R343	SSmDrKMAELRGEMER	M3(Ox); R5(CMA)	3	667.31	34.81	1.9

120	E1BM70	Coiled-coil domain-containing protein 39	K506	TLEEKKSALcLLETQIkK	C10(Cam); K17(HHE)	5	450.06	34.34	3.8
121	Q2TA16	Coiled-coil domain-containing protein 65	K33	LLAEEEMAkKKER	K9(CML)	3	544.96	52.01	2.0
122	Q2UVX4	Complement C3	K586, K598	NkLTQRKIWDVVEk	K2(AL); K14(AL)	4	461.02	49.64	2.7
123	A2VDR8	Conserved oligomeric Golgi complex subunit 7	K91	EQmILVkEDIKK	M3(Ox); K7(HHE)	3	535.31	30.55	2.1
124	A7MB90*	CSNK1G2 protein	R179	rQHAIHIIDFGLAK	R1(THP)	4	441.50	26.21	2.9
125	E1BB52	Cyclin-dependent kinase 13	K409	RSAkSRSR	K4(FL)	2	488.28	36.15	1.7
126	A5PK21	Cytoskeleton-associated protein 2-like	K28, K33	LkCQNTkPYLK	K2(AL); K7(AL)	3	473.92	30.25	2.0
127	F1MQY1*	DNA ligase	K145	EWYHIKCMFEkLER	K11(Propanal)	3	651.32	41.36	2.4
128	E1BPY8*	DNA polymerase	K1540	ELLQkRQQK	K5(Lac)	3	498.94	46.39	2.3
129	E1BJF4*	DNA polymerase	R682, K688	RSNmPKLGGrSGFGEk	M4(Ox); R10(CMA); K16(CEL)	3	622.98	45.64	2.0
130	Q5E954	DnaJ homolog subfamily A member 1	K130	LALQkNVICDKCEGR	K5(AL)	3	577.97	35.39	2.3
131	A6QPP7*	ELA2 protein	R67	QErTRQMFR	R3(CMA)	3	437.22	23.61	2.5
132	P81623	Endoplasmic reticulum resident protein 29	K205	LIEKNKmSDGkK	M7(Ox); K11(CML)	3	488.93	28.39	2.1
133	P17322	Endothelin-1	K94	RCQCASQTDKkCWNFcQAGK	K11(CML); C16(Cam)	3	807.35	31.45	2.1
134	Q17QK4*	Epoxide hydrolase 2, cytoplasmic	K471	NmDkNWEWGFKGSGR	M2(Ox); K4(Nonanal)	3	651.32	41.82	2.4
135	Q5E997	F-actin-capping protein subunit alpha-2	K273, K278	TKIDWNkILSYkIGK	K7(FL); K12(FL)	4	466.51	29.87	2.2
136	Q3ZBT2	F-box only protein 9	K83	EEkARELFLK	K3(FL)	2	645.86	30.40	1.9
137	P43249	G protein-coupled receptor kinase 5	R47, Y53	rTIDRDyCSLCDK	R1(ACR); Y7(Ox)	4	415.69	34.22	3.2
138	A5D7A2*	GARS protein	K229	ADHLLKAHLQKLMSDkK	K16(CML)	4	509.29	32.67	3.1
Nr.	**Accession**	**Description**	**Mod. site protein**	**Sequence**	**Mod. site peptide** [a)]	**z**	***m/z***	**t_R [min]**	**Xcorr**
139	Q863C3	Gastrin-releasing peptide	H20	GNhWAVGHLmGK	H3(Pent); M10(Ox)	3	469.57	75.72	1.7
140	A6QLU1	Glycerol-3-phosphate dehydrogenase, mitochondrial	K458, K473	AHNLkAGPSRTVGLFLQGGk	K5(OMA); K20(OMA)	3	732.38	40.79	2.3
141	Q3T0T7	GTP-binding protein SAR1b	K24, K27	kTGkLVFLGLDNAGK	K1(FL); K4(AL)	3	544.31	44.00	2.1
142	E1BN71*	Guanylate cyclase	R540, R545	KrIELTrEVLFELK	R2(CMA); R7(CMA)	4	473.28	27.62	3.1
143	E1BN71*	Guanylate cyclase	R540, R545	rIELTrEVLFELK	R1(CMA); R6(CMA)	4	441.25	29.04	2.6
144	Q76BS1	Hepatocyte growth factor	C504	QcFPSRNKDLK	C2(Pent)	3	473.92	46.82	1.8

145	P10103	High mobility group protein B1	K75	EmkTYIPPKGETKK	M2(Ox); K3(Heptanal)	4(3)	567.04	28.98	3.3
146	E1BEB3*	Histone acetyltransferase	R1115	LASQDVLRCrSDSKR	R10(Octanal)	3	615.35	45.90	2.4
147	Q3T022	Histone deacetylase complex subunit SAP18	K18, K24	KEPEkPIDREkTcPLLLR	K5(FL); K11(FL); C13(Cam)	4	570.32	42.80	2.6
148	A6H709*	HSPC321 protein	R368	KKLEEAASrAAEEEK	R9(Propanal)	3	576.98	30.43	2.7
149	F1N1G7*	Kinesin-like protein	R720	QISSLrDEVEAK	R6(5-oxo pentanoic acid)	3	491.59	29.77	2.4
150	A6H7H2*	KLC1 protein	H147	KhLEFMNQLKK	H2(HHE)	3	510.62	27.11	2.3
151	A5HLY3*	Lactoferrin	P88	EApQTHYYAVAVVK	P3(GSA)	3	531.27	27.96	3.6
152	A5HLY3*	Lactoferrin	T90	EAPQtHYYAVAVVKK	T5(Ox)	4	426.23	22.95	2.6
153	P80025	Lactoperoxidase	R537	CrDHGMPGYNSWR	R2(Glarg)	3	540.23	30.05	2.5
154	Q24K06	Leucine-rich repeat-containing protein 10	K109, R115	QLCILYLGNNkLcDLPrELSLLQNLR	K11(CML); C13(Cam); R17(CMA)	4	804.68	49.19	2.9
155	Q32KP2	Leucine-rich repeat-containing protein 23	K202	LkNLFLAQNmLKK	K2(OMA); M10(Ox)	3	550.31	28.02	2.0
156	F1MCA7	Leucine-rich repeat-containing protein 7	K926	GVISISkSTERLSPLmKDIK	K7(FL); M16(Ox)	5	450.06	34.06	4.3
157	P80513	Mesencephalic astrocyte-derived neurotrophic factor	K150	INELMPKYAPkAASSR	K11(Pentanal)	3	615.35	45.86	2.8
158	F1MEW3*	Microtubule-associated protein	K1011	ELTVAkDASPVMAEKAEK	K6(CML)	3	659.01	46.61	2.3
**Nr.**	**Accession**	**Description**	**Mod. site protein**	**Sequence**	**Mod. site peptide [a)]**	**z**	***m/z***	**t_R [min]**	**Xcorr**
159	Q0III0	Myb/SANT-like DNA-binding domain-containing protein 3	K71, K72	QLkkCWENIK	K3(GL); K4(AL)	3	473.91	46.71	2.0
160	P17667	Myogenic factor 5	L135,Q136	YIESlqELLR	L5(+14 Ox); Q6(+14 Ox)	3	431.23	56.50	3.2
161	Q8SPU7	Neuronal acetylcholine receptor subunit alpha-5	H440	WANIIVPIhIGNENK	H9(Octenal)	3	615.35	45.94	2.6
162	Q2KJB5	Nostrin	K172	QKLLNkLKK	K6(FL)	2	570.88	52.55	1.9
163	Q2KJB5	Nostrin	K305	SkLLRLQK	K2(AL)	2	514.33	52.61	2.4
164	A6QLD2	Olfactomedin-like protein 2B	K116, K126	LQkLREADSRDLk	K3(FL); K13(FL)	3	543.30	20.58	2.7
165	E1BBD8*	Oxysterol-binding protein	R808	FFRrQTDSSGK	R4(Argpyr)	3	470.23	59.55	2.7
166	A0JNA8	PAX-interacting protein 1	K649	LmAYLAGAkYTGYLCR	M2(Ox); K9(CML)	3	623.31	37.29	2.5
167	Q1LZ80	PAXIP1-associated glutamate-rich protein 1	R210	LDKVLSDmKrHK	M8(Ox); R10(MGH)	3	513.95	43.45	2.4
168	Q3ZBL5	Peptidyl-tRNA hydrolase 2, mitochondrial	K14	mILVVRNDLkmGKGK	M1(Ox); K10(FL); M11(Ox)	3	588.00	28.75	1.9
169	Q9TUM6/ F1MQB0*	Perilipin 2	K289	IQDAQDKLYLSWLEWk	K16(HNE)	3	731.39	51.50	3.2

170	A6QNW3	PIGR protein	K256	kNGGACNVVINTLGKK	K1(CML)	3	558.64	28.56	2.5
171	A6QNW3	PIGR protein	K256	kNGGACNVVINTLGK	K1(CML)	3	515.94	31.85	2.5
172	A4IFL4*	PPARD protein	R258	cQCTTVETVrELTEFAK	C1(Cam); R10(CMA)	3	691.66	19.17	2.4
173	Q3B7L8	Pre-mRNA-splicing factor RBM22	K311	SQAARGkEKEK	K7(Hex)	3	465.25	25.06	1.8
174	Q3T090	Pre-rRNA-processing protein TSR2 homolog	K119	AALKEMASLITQRkcK	K14(CML); C15(Cam)	4	477.27	35.58	3.1
175	Q3SZH6	Probable tubulin polyglutamylase TTLL9	R447, R451	EQLrQLFrSLQGQKK	R4(Argpyr); R8(Argpyr)	4	505.54	32.64	2.5
176	Q0P5F2	Proteasome assembly chaperone 1	K54	QTkTTLEVSLLEK	K3(HHE)	3	535.31	30.49	2.1
177	Q1RMK1	Protein FAM92B	R118	FKSVrNNEIK	R5(GSA)	2	596.32	39.88	1.5
178	A7YY45	Protein IMPACT	K198	QVkMVLAK	K3(Lac)	3	414.23	31.34	2.2
179	Q3MHH1	Protein SHQ1 homolog	K398, K399	SkkLAALAEALKK	K2(GLAP); K3(Pyrraline)	3	529.98	38.44	2.2

Nr.	**Accession**	**Description**	**Mod. site protein**	**Sequence**	**Mod. site peptide** [a)]	**z**	***m/z***	**t_R [min]**	**Xcorr**
180	P42201	P-selectin	K66	KINNKWTWVGTkK	K12(FL)	3	544.30	26.34	2.7
181	A6QNR1	Ribosomal RNA processing protein 36 homolog	K140, K141	ELVkkQLKK	K4(GL); K5(GL)	2	645.39	27.04	1.8
182	Q7YRD0	RNA-binding protein PNO1	K122	SRNVEIRTckETK	C9(Cam); K10(Tridecanal)	3	601.01	38.98	2.4
183	Q6Q137	Septin-7	K219	KQIMKEIQEHkIK	K11(GLAP)	4	441.25	29.46	3.4
184	E1BP17*	Serine/threonine-protein kinase PLK	K24	MCEQALGkAcGGDSKK	K8(Propanal); C10(Cam)	3	574.94	26.82	2.7
185	A2I7N0	Serpin A3-4	K167	TQGKIEELFkDLSPR	K10(AL)	3	601.66	48.04	2.4
186	A5PKM3*	SPTLC2 protein	R302	LLKDAIVYGQPrTR	R12(Glarg)	3	557.32	48.50	1.7
187	O97594	Structural maintenance of chromosomes protein 3	K393	EERDkWIKK	K5(Hex)	3	465.25	25.01	2.0
188	P0C1G6	Switch-associated protein 70	K529, K537	LEmAAkmTKSWKDk	M3(Ox); K6(FL); M7(Ox); K14(FL)	3	585.62	46.49	2.1
189	Q3T0I4	THO complex subunit 4	R202	GGMTrNRGSGGFGGGGGTR	R5(CMA)	3	599.62	44.58	2.2
190	Q3T086	Trafficking protein particle complex subunit 6A	K67	LPRETLTFREELDILk	K16(FL)	3	667.71	39.89	2.0
191	E1BLA4*	Transmembrane 9 superfamily member	E132, K136	eDQKkKLDFLK	E1(+14 Ox); K5(+14 Ox)	3	473.92	30.37	2.8
192	Q08DV9	Transmembrane protein 131-like	E1164, C1167	KIHKAAKeDGcSEK	E8(+14 Ox); C11(Ox)	3	525.26	22.06	2.3
193	Q5E9T5	Transmembrane protein 35A	K34	LSkDAYSEmKRAYK	K3(Hex); M9(Ox)	3	623.31	33.87	1.9

Nr.	Accession	Description	Mod. site protein	Sequence	Mod. site peptide [a)]	z	*m/z*	t_R [min]	Xcorr
194	Q0VC16	Transport and Golgi organization protein 1 homolog	K1239	QNMILSDEAIkFKDK	K11(Octenal)	3	636.02	35.55	2.5
195	Q0VC16	Transport and Golgi organization protein 1 homolog	K1241	QNMILSDEAIKFkDK	K13(Octenal)	3	636.02	35.56	2.8
196	Q0VC16	Transport and Golgi organization protein 1 homolog	K1460	LRASmSTkcNLEDQIKK	M5(Ox); K8(Pentadecanal); C9(Cam)	5(4)	456.29	34.31	4.7
197	A5D980*	Tyrosine-protein phosphatase non-receptor type	R407	LRNLQVSPLENENLIr	R16(MGH)	3	654.70	52.86	2.7
**Nr.**	**Accession**	**Description**	**Mod. site protein**	**Sequence**	**Mod. site peptide** [a)]	**z**	***m/z***	**t_R [min]**	**Xcorr**
198	P10568	Unconventional myosin-Ia	K855	EKLcASELFk	C4(Cam); K10(FL)	3	418.22	27.95	2.4
199	Q1LZE8	UPF0415 protein C7orf25 homolog	K41, K47	LCSkLKAELkFLQK	K4(AL); K10(OMA)	3	588.33	26.09	1.7
200	Q2T9M0	UPF0602 protein C4orf47 homolog	K190	LLkGTAFK	K3(OMA)	2	475.27	22.88	1.7
201	Q5E9A6	Vacuolar protein-sorting-associated protein 25	K84	kGNLEWLDKNK	K1(HHE)	3	486.94	42.25	2.9
202	Q3SZP7/ Q5E9Z3*	Villin 1	K309	kEAMNQALNFIK	K1(HHE)	3	507.61	30.23	2.2
203	P80012	von Willebrand factor	H54, R57	hENrcVALER	H1(ACR); R4(ACR); C5(Cam)	3	465.89	19.26	1.6
204	Q32LB0	WD repeat-containing protein 70	K167, K181	kIPDSHEITIKHGTk	K1(FL); K15(FL)	3	587.32	98.07	2.0

[a)] Abbreviations used for modifications: Cam - carbamidomethylation of Cys, Ox - oxidation of Met, Cys and Tyr. Hex - hexosylation of Lys, Lac lactosylation of Lys, CEL - carboxyethylation of Lys, CML - carboxymethylation of Lys, CMA carboxymethylation of Arg, FL - formyl-Lys, AL - acetyl-Lys, GL - glycerinyl-Lys, GLAP - glyceraldehyde-derived pyridinium on Lys, OMA - Lys modified by oxalic acid monolysinylamide, Glarg - glyoxal-derived hydroimidazolium on Arg, MGH - methylglyoxal-derived hydroimidazolones on Arg, THP - tetrahydropyrimidine on Arg, Argpyr – argpyrimidine on Arg, GSA - glutamic semialdehyde at Pro or Arg, Pent - pentenal, ACR - acrolein, CRO - crotonaldehyde, OHE - 4-oxo-2-hexenal, HNE - 4-hydroxy-2-nonenal and HHE - 4-hydroxy-2-hexenal, +14 - carbonylation of Glu, Leu, Gln, Lys or Val.

Table 3. Overview of identified protein modification sites.

Accession	Description	Amadori modification site	AGE modification site	Carbonylation/ oxidation site	LMW carbonyl-protein adduct site	Unique modification site
P00711	Alpha lactalbumin	K5, K98	K98	T86	K93	K5, T86, K93, K98
P02754	Beta-lactoglobulin	K47, K75, K77, K83, K91, K100, K101, K135, K138, K141	K69, K70, K77, K91, K135, K141	K69, C66		K47, C66, K69, K70, K75, K77, K83, K91, K100, K101, K135, K138, K141
P02662	Alpha-S1-casein	K7, K34, K36, K132	K7	P5, P29		P5, K7, P29, K34, K36, K132
P02663	Alpha-S2-casein	K32, K41, K158, K165, K173, K181, K188, K191, K199	K32, K41, K152, K158, K199, R205	C36, C40, T38, K41		K32, C36, T38, C40, K41, K152, K158, K165, K173, K181, K188, K191, K199, R205
P02666	Beta-casein	K176	K176	P186		K176, P186
P02668	Kappa-casein	K21, K24, K46, K63, K86		P99, P101		K21, K24, K46, K63, K86, P99, P101
P02769	Serum albumin	K232, K275		C476		K232, K275, C476
P80195	Glycosylation-dependent cell adhesion molecule 1	K71, K73, K116			R109	K71, K73, R109, K116
Q2KJ46	26S proteasome non-ATPase regulatory subunit 3				K503	K503
Q3T0X6	40S ribosomal protein S16		K33, R37			K33, R37
Q58DW5	60S ribosomal protein L5		K241, K242			K241, K242
Q9GLE5	72 kDa type IV collagenase				K42	K42
Q9TT92	A disintegrin and metalloproteinase with thrombospondin motifs 5		R144			R144
A4IFD0	Adenylate kinase isoenzyme 5		K389			K389
Q3SZF2	ADP-ribosylation factor 4				R19	R19

Q3MHM0*	Amyloid beta (A4) protein-binding, family B, member 1 interacting protein		K282, K291			K282, K291
Q3SWX7	Annexin A3		K169, K177			K169, K177
Q7YRZ2	Aprataxin		K61, K62			K61, K62
Q29RI0	Atypical kinase COQ8A, mitochondrial		K612			K612
Q5I597	Betaine--homocysteine S-methyltransferase 1			Q142, L145, V149		Q142, L145, V149
Q32S26	Bromodomain-containing protein 2		K329, K342			K329, K342
Q3SWX5	Cadherin-6		R684			R684
Q29RI6	Calcium-binding protein 39			H153		H153
Q58CW6	Calcium-responsive transcription factor			K347		K347
Q08DY9	Caspase-3		K197, R201			K197, R201
Q29RT4	Cell division cycle-associated protein 2		R821			R821
Q32LN4*	Coiled-coil domain containing 19	K390				K390
A0JNH6	Coiled-coil domain-containing protein 102A		R343			R343
E1BM70	Coiled-coil domain-containing protein 39			K506		K506
Q2TA16	Coiled-coil domain-containing protein 65		K33			K33
Q2UVX4	Complement C3		K586, K598			K586, K598
A2VDR8	Conserved oligomeric Golgi complex subunit 7			K91		K91
A7MB90*	CSNK1G2 protein		R179			R179
E1BB52	Cyclin-dependent kinase 13		K409			K409
A5PK21	Cytoskeleton-associated protein 2-like		K28, K33			K28, K33
F1MQY1*	DNA ligase				K145	K145
E1BPY8*	DNA polymerase	K1540				K1540

E1BJF4*	DNA polymerase	R682, K688			R682, K688
Q5E954	DnaJ homolog subfamily A member 1	K130			K130
A6QPP7*	ELA2 protein	R67			R67
P81623	Endoplasmic reticulum resident protein 29	K205			K205
P17322	Endothelin-1	K94			K94
Q17QK4*	Epoxide hydrolase 2, cytoplasmic			K471	K471
Q5E997	F-actin-capping protein subunit alpha-2	K273, K278			K273, K278
Q3ZBT2	F-box only protein 9	K83			K83
P43249	G protein-coupled receptor kinase 5		R47, Y53		R47, Y53
A5D7A2*	GARS protein	K229			K229
Q863C3	Gastrin-releasing peptide		H20		H20
A6QLU1	Glycerol-3-phosphate dehydrogenase, mitochondrial	K458, K473			K458, K473
Q3T0T7	GTP-binding protein SAR1b	K24, K27			K24, K27
E1BN71*	Guanylate cyclase	R540, R545			R540, R545
Q76BS1	Hepatocyte growth factor		C504		C504
P10103	High mobility group protein B1			K75	K75
E1BEB3*	Histone acetyltransferase			R1115	R1115
Q3T022	Histone deacetylase complex subunit SAP18	K18, K24			K18, K24
A6H709*	HSPC321 protein			R368	R368
F1N1G7*	Kinesin-like protein			R720	R720
A6H7H2*	KLC1 protein		H147		H147
A5HLY3*	Lactoferrin		P88, T90		P88, T90
P80025	Lactoperoxidase	R537			R537
Q24K06	Leucine-rich repeat-containing protein 10	K109, R115			K109, R115
Q32KP2	Leucine-rich repeat-containing protein 23	K202			K202

P80513	Mesencephalic astrocyte-derived neurotrophic factor				K150	K150
F1MEW3*	Microtubule-associated protein		K1011			K1011
Q0III0	Myb/SANT-like DNA-binding domain-containing protein 3		K71, K72			K71, K72
P17667	Myogenic factor 5			L135,Q136		L135,Q136
Q8SPU7	Neuronal acetylcholine receptor subunit alpha-5				H440	H440
Q2KJB5	Nostrin		K172, K305			K172, K305
A6QLD2	Olfactomedin-like protein 2B		K116, K126			K116, K126
E1BBD8*	Oxysterol-binding protein		R808			R808
A0JNA8	PAX-interacting protein 1		K649			K649
Q1LZ80	PAXIP1-associated glutamate-rich protein 1		R210			R210
Q3ZBL5	Peptidyl-tRNA hydrolase 2, mitochondrial		K14			K14
Q9TUM6/ F1MQB0*	Perilipin 2			K289		K289
A6QNW3	PIGR protein		K256			K256
A4IFL4*	PPARD protein		R258			R258
Q3B7L8	Pre-mRNA-splicing factor RBM22	K311				K311
Q3T090	Pre-rRNA-processing protein TSR2 homolog		K119			K119
Q3SZH6	Probable tubulin polyglutamylase TTLL9		R447, R451			R447, R451
Q0P5F2	Proteasome assembly chaperone 1			K54		K54
Q1RMK1	Protein FAM92B			R118		R118
A7YY45	Protein IMPACT	K198				K198
Q3MHH1	Protein SHQ1 homolog		K398, K399			K398, K399
P42201	P-selectin		K66			K66
A6QNR1	Ribosomal RNA processing protein 36 homolog		K140, K141			K140, K141
Q6Q137	Septin-7		K219			K219

E1BP17*	Serine/threonine-protein kinase PLK				K24	K24
A2I7N0	Serpin A3-4				K167	K167
A5PKM3*	SPTLC2 protein		R302			R302
O97594	Structural maintenance of chromosomes protein 3	K393				K393
P0C1G6	Switch-associated protein 70		K529, K537			K529, K537
Q3T0I4	THO complex subunit 4		R202			R202
Q3T086	Trafficking protein particle complex subunit 6A		K67			K67
E1BLA4*	Transmembrane 9 superfamily member			E132, K136		E132, K136
Q08DV9	Transmembrane protein 131-like			E1164, C1167		E1164, C1167
Q5E9T5	Transmembrane protein 35A	K34				K34
Q0VC16	Transport and Golgi organization protein 1 homolog				K1239, K1241, K1460	K1239, K1241, K1460
A5D980*	Tyrosine-protein phosphatase non-receptor type		R407			R407
P10568	Unconventional myosin-Ia		K855			K855
Q1LZE8	UPF0415 protein C7orf25 homolog		K41, K47			K41, K47
Q2T9M0	UPF0602 protein C4orf47 homolog		K190			K190
Q5E9A6	Vacuolar protein-sorting-associated protein 25			K84		K84
Q3SZP7/ Q5E9Z3*	Villin 1			K309		K309
P80012	von Willebrand factor			H54, R57		H54, R57
Q32LB0	WD repeat-containing protein 70		K167, K181			K167, K181

* non-reviewed protein.

Table 4. CHH-derivatized compounds identified in the final product of each milk drink by static ESI-MS/MS relying on specific CHH fragments and molecular formula. An x indicates that the species was confirmed by its fragment spectrum and specific CHH-ions within the sample.

m/z [M-CHH]	elemental composition	elemental composition analyte	elemental mass	proposed compound	Ch [b] (3.5% UHT)	S [b] (3.5%, UHT)	Co [b] (1.5%, P)	V [b] (1.5%, UHT)
314.1500	$C_{17}H_{20}O_3N_3$	C_3H_4O	56.0257	acrolein	x	noMSMS [c]	x	noMSMS [c]
316.1656	$C_{17}H_{22}O_3N_3$	C_3H_6O	58.0413	propanal	x	x	x	x
318.1448	$C_{16}H_{20}O_4N_3$	$C_2H_4O_2$	60.0205	2-hydroxyethanal	x	x	x	x
330.1812	$C_{18}H_{24}O_3N_3$	C_4H_8O	72.0569	butanal [a]	x	x	x	x
332.1604	$C_{17}H_{22}O_4N_3$	$C_3H_6O_2$	74.0361	hydroxypropanal [a]	x	x	x	x
344.1968	$C_{19}H_{26}O_3N_3$	$C_5H_{10}O$	86.0725	pentanal	x	x	x	x
346.1760	$C_{18}H_{24}O_4N_3$	$C_4H_8O_2$	88.0517	4-hydroxybutanal [a]	x	x	x	x
348.1553	$C_{17}H_{22}O_5N_3$	$C_3H_6O_3$	90.0310	2,3-dihydroxypropanal [a]	x	x	x	x
358.2122	$C_{20}H_{28}O_3N_3$	$C_6H_{12}O$	100.0879	hexanal	x	x	x	x
360.1914	$C_{19}H_{26}O_4N_3$	$C_5H_{10}O_2$	102.0671	3-hydroxypentanal [a]	x	x	x	x
372.2279	$C_{21}H_{30}O_3N_3$	$C_7H_{14}O$	114.1036	heptanal	x	x	x	x
374.1705	$C_{19}H_{24}O_5N_3$	$C_5H_8O_3$	116.0462	5-oxo-pentanoic acid	noMS/MS [c]	noMS/MS [c]	x	noMS/MS [c]
384.2276	$C_{22}H_{30}O_3N_3$	$C_8H_{14}O$	126.1033	octenal	x	noMS/MS [c]	x	noMS/MS [c]
386.2433	$C_{22}H_{32}O_3N_3$	$C_8H_{16}O$	128.1190	octanal	x	x	x	x
398.2432	$C_{23}H_{32}O_3N_3$	$C_9H_{16}O$	140.1189	nonenal	x	x	x	x
400.2588	$C_{23}H_{34}O_3N_3$	$C_9H_{18}O$	142.1345	nonanal	x	x	x	x
428.2933	$C_{25}H_{38}O_3N_3$	$C_{11}H_{22}O$	170.1690	undecanal	x	x	x	x
456.3247	$C_{27}H_{42}O_3N_3$	$C_{13}H_{26}O$	198.2004	tridecanal	x	x	x	x
470.3404	$C_{28}H_{44}O_3N_3$	$C_{14}H_{28}O$	212.2161	tetradecanal	x	noMS/MS [c]	noMS/MS [c]	noMS/MS [c]
484.3532	$C_{29}H_{46}O_3N_3$	$C_{15}H_{30}O$	226.2289	pentadecanal	x	x	x	only *m/z* 244 [d]
573.2458	$C_{30}H_{33}O_6N_6$	$C_2H_2O_2$	58.0051	glyoxal [a]	x	x	x	x

[a] Modifications were not considered for targeted analysis as the mass shifts resulting from their reaction with amino acids overlap with previously identified modifications present in milk. Butanal leads to the same mass shift as MDA and MGH, hydroxypropanal overlaps with ACR, 4-hydroxybutanal with CRO, 2,3-dihydroxypropanal with CEL/CEA, 3-hydroxypentanal with pentenal, 2-hydroxyethanal (glycolaldehyde) with AL, and glyoxal with CML/CMA.[b] Abbreviations used for milk drinks: Ch stands for chocolate, S for strawberry, V for vanilla, and Co for cocoa milk drink. All samples were collected after the second thermal processing, either P for pasteurization or UHT for ultra-high temperature treatment.[c] No MS/MS indicates that the precursor was detected in the survey scan, but no MS/MS was recorded.[d] Only *m/z* 244 indicates that the corresponding MS/MS spectrum contained only one specific CHH-fragment.
